# Observation of the application effect of low-volume polyethylene glycol electrolyte lavage solution (PEG-ELS) combined with ascorbic acid tablets in bowel preparation for colonoscopy in hospitalized patients

**DOI:** 10.3389/fonc.2023.1038461

**Published:** 2023-04-14

**Authors:** Le-Can Wu, En-Dian Zheng, Hao-Yue Sun, Xi-Zhou Lin, Ju-Yi Pan, Xiao-Xiao Lin

**Affiliations:** Department of Gastroenterology, Wenzhou People’s Hospital, The Wenzhou Third Clinical Institute Affiliated to Wenzhou Medical University, Wenzhou, Zhejiang, China

**Keywords:** polyethylene glycol, bowel preparation, colonoscopy, colorectal cancer, ascorbic acid

## Abstract

**Background:**

This study explored the effectiveness and safety of low-volume polyethylene glycol electrolyte lavage solution (PEG-ELS) combined with ascorbic acid tablets (PEG-ELS/Asc) in bowel preparation for a colonoscopy.

**Methods:**

A total of 240 hospitalized patients who underwent a colonoscopy in Wenzhou People’s Hospital, Wenzhou Third Clinical College of Wenzhou Medical University from July 2020 to June 2022 were randomly divided into two groups, with 120 patients each. All of the participants were given a low-residue or residue-free diet one day before the examination and fasted after dinner (completed before 18:00) the day before the examination. The 2-L PEG-ELS/Asc group took 2-L PEG-ELS plus 10 g ascorbic acid tablets once orally, while the 3-L PEG-ELS group took 3-L PEG orally on several occasions. The primary endpoint was the achievement of preparation adequacy and an overall colon cleansing score of ≥6, both assessed by blinded investigators using the Boston Bowel Preparation Scale (BBPS). The bowel cleansing effect, polyp detection rate, adverse reaction rate, oral drug tolerance rate, renal function, and electrolyte level changes were also compared between the two patient groups.

**Results:**

There were no significant differences in the success rate of bowel preparation, the detection rate of polyps, or the adverse reaction rate between the two groups (*P* > 0.05). The tolerance rate of bowel preparation in the 2-L PEG-ELS/Asc group was significantly higher than that in the 3-L PEG-ELS group (93.3% vs. 80.23%) (*P* < 0.05). The levels of creatinine, serum potassium, serum sodium, and serum chlorine of the two groups before and after bowel preparation were within the normal range. In addition, the intestinal cleaning effect of the two preparation schemes for the hospitalized patients with diabetes and constipation is worse than that of those without these conditions (*P* < 0.05).

**Conclusion:**

The effectiveness and safety of using 2-L PEG-ELS/Asc in bowel preparation for a colonoscopy in hospitalized patients were not inferior to using 3-L PEG-ELS. For patients with diabetes and constipation, the cleansing effect of the two bowel preparation options was not very satisfactory, and further clinical research is needed in this regard.

## 1 Background

With changes in people’s way of life and dietary structure, there has been an upward trend in the incidence of colorectal cancer. According to global cancer data, colorectal cancer ranks third in the incidence of malignant tumors (1). At present, a colonoscopy is effective for the early detection and prevention of colorectal cancer, and adequate and effective bowel preparation is an important prerequisite for a successful colonoscopy. Because of its good safety and effectiveness, polyethylene glycol electrolyte lavage solution (PEG-ELS) is generally recommended as the preferred method for bowel cleansing before a colonoscopy, based on relevant guidelines (2, 3). However, the large intake of liquid (3–4 L) and poor taste of the standard PEG-ELS option greatly reduces the tolerance and compliance of patients, thus affecting the quality of bowel preparation.

Ascorbic acid, also known as vitamin C, has a laxative effect and can reduce PEG-ELS usage as an adjuvant drug. In addition, its relatively acceptable taste can improve the patient’s tolerance and compliance to some degree (2, 4, 5). Recently, a low-volume 2-L PEG-ELS plus ascorbic acid (2-L PEG-ELS/Asc) bowel cleansing option was developed and applied in Western countries and Japan. Previous studies have shown that bowel preparation for colonoscopy in outpatients with 2-L PEG-ELS/Asc is effective, well-tolerated, and safe (4, 5). As for hospitalized patients, because they have low activity levels and slow gastrointestinal motility, the bowel cleansing effect is unsatisfactory. Although several interventions have been implemented, such as various purgatives, split dose, and alterations in timing of their administration on bowel cleansing, inadequate bowel preparation in hospitalized patients undergoing colonoscopy still exists (6–8).

Currently, PEG-ELS/Asc for bowel preparation is mostly focused on outpatients, and there are fewer studies on the use of PEG-ELS/Asc for bowel preparation in hospitalized patients (9).The study sample sizes of relevant research have been small and present problems, e.g., uneven research quality and regional differences. Therefore, this study aimed to explore the effectiveness and safety of low-volume PEG-ELS (containing PEG 4000) combined with ascorbic acid tablets (PEG-ELS/Asc) in bowel preparation for a colonoscopy in hospitalized patients.

## 2 Data and method

### 2.1 General data

Hospitalized patients who underwent a colonoscopy in Wenzhou People’s Hospital, Wenzhou Third Clinical College of Wenzhou Medical University from July 2020 to June 2022 were selected as study participants. The exclusion criteria were as follows: 1) patients not aged between 18 and 65 years; 2) pregnant women; 3) patients with inflammatory bowel disease, organ failure, gastrointestinal perforation, gastrointestinal bleeding, and intestinal obstruction; 4) individuals who were taking non-steroidal anti-inflammatory drugs, such as aspirin; 5) patients who refused to participate in the trial; 6) patients who had undergone multiple abdominal surgeries and obvious intestinal adhesion; 7) individuals who were allergic to the drugs used in this trial. Using the random number table method, the included participants were arranged into 2-L PEG-ELS/Asc group and 3-L PEG-ELS group, respectively. This study was approved by the ethics committee of the authors’ hospital. All of the patients signed the informed consent forms to confirm their inclusion in the research.

### 2.2 Methods

All participants were given a low-residue or residue-free diet one day before the examination and fasted after dinner (completed before 18:00) the day before the examination. Polyethylene glycol electrolyte powder (Hygecon Zhengqing™) was purchased from Jiangxi Hygecon Pharmaceutical Co., Ltd.; each box comprised A, B, and C packs, where A included 0.74 g potassium chloride and 1.68 g sodium bicarbonate, B included 1.46 g sodium chloride and 5.68 g sodium sulfate, C included 60 g polyethylene glycol 4000. Ascorbic acid tablets were purchased from Hainan Pharmaceutical Co., Ltd., and each tablet comprised 0.1 g of ascorbic acid.

In the 2-L PEG-ELS/Asc group, one box of polyethylene glycol electrolyte powder was prepared into a 1-L cathartic solution. After the Polyethylene glycol electrolyte powder had been dissolved, 5.0 g ascorbic acid tablets were added, fully rolled, and stirred to dissolve. The single oral administration of 2-L PEG-ELS, combined with 10.0 g ascorbic acid tablets, was adopted in the 2-L PEG-ELS/Asc group. Two liters of cathartic solution plus 20 ml simethicone were administered orally 4–6 h before the examination on the same day, 250 ml every 15 min, and finished within 2 h. In the 3-L PEG-ELS group, the patients received 3-L PEG-ELS orally several times. One box of polyethylene glycol electrolyte powder was prepared into a 1-L cathartic solution, which was taken at 21:00 the day before the examination, 250 ml every 15 min, and finished within 1 h. The remaining 2 L of cathartic fluid was taken 4–6 h before the examination. The last 1 L of the cathartic solution was added with 20 ml of simethicone, taken 250 ml every 15 min, and finished within 2 h. After taking the medication, both groups were instructed to exercise appropriately to promote emptying. All patients started taking the medication with appropriate exercise, walking every 15 minutes for 5 minutes. Patients who could not walk performed clockwise abdominal massage: 10 minutes/time, 30 minutes/time interval.

The colonoscopy was performed by two expert endoscopists with more than 5 years of experience. An Olympus (H290) endoscope was selected to conduct the procedure. In addition, the two endoscopists were well informed of Boston Bowel Preparation Scale (BBPS) scoring system through training programs. An endoscopy physician who was familiar with BBPS scored the bowel cleansing. The endoscopy physician was unaware of the patient grouping, thereby ensuring the fairness of the grade evaluation and reducing the judgment error caused by human factors.

### 2.3 Observation indicators

(1) Bowel cleansing score. The BBPS was used in this study (10), and the entire colon was divided into three segments, i.e., the right colon (the ileocecal part plus the ascending colon), transverse colon (the hepatic flexure plus the transverse colon plus the splenic flexure), and the left colon (the descending colon plus the sigmoid colon plus the rectum).

Each segment was scored as follows: 0 (unprepared colon segment with mucosa not seen due to solid stool that cannot be cleared); 1 (portion of mucosa of the colon segment seen, but other areas of the colon segment not well seen due to staining, residual stool and/or opaque liquid); 2 (minor amount of residual staining, small fragments of stool and/or opaque liquid, but mucosa of colon segment seen well); 3 (entire mucosa of colon segment seen well, with no residual staining, small fragments of stool, or opaque liquid). The total bowl cleansing score was that of the three intestinal segments combined (0–9). A total score ≥ 6 or scores of 2 or 3 for all colon segments indicated adequate bowel preparation, and any segment colon score = 0 or 1 reflected inadequate bowel preparation (11). According to the total BBPS score, the overall preparation status was classified as following: excellent (BBPS 8–9), good (BBPS 6–7), poor (BBPS 3–5) or very poor (BBPS 0–2). These scores were used to compare the difference in bowel preparation quality between the two groups.

(2) Detection rates of polyps and adenomas. The detection rate of polyps was the proportion of one or more polyps detected during the colonoscopy. The detection rate of adenoma was the proportion of one or more adenomas detected *via* colonoscopy (the detection rate of polyps or adenomas = the number of cases with polyps or adenomas detected/the total number of cases × 100%).

(3) Evaluation of adverse reactions and tolerance. Patients were investigated for adverse reactions and tolerance following the completion of bowel preparation. Adverse reactions included nausea, vomiting, abdominal distension, abdominal pain, and dizziness.

The evaluation criteria of tolerance were as follows: degree I – completely tolerable, willing to accept bowel preparation again; degree II – with slight nausea, abdominal distension, abdominal pain, dizziness, and other discomforts, but tolerable; degree III – experienced severe reactions following the oral administration of drugs, intolerable, and the patient refused to undergo such an examination again; degrees I + II were considered “tolerable,” and degree III was considered “intolerable.”

(4) Assessment of renal function damage and electrolyte disturbance. The creatinine and electrolyte levels of patients before and after bowel preparation were detected to assess the changes in the renal function and electrolyte levels of the participants in the two groups after bowel preparation.

In this study, the primary outcome indicator was the bowel preparation success rate, defined as the number of patients with a total BBPS score ≥6 per group/number of patients per group × 100%. The secondary outcome indicator was the bowel preparation tolerance rate, defined as the number of patients in each group with I+II tolerance/number of patients in each group × 100%. The efficiency of bowel preparation was mainly assessed by the success rate of bowel preparation, and the safety was mainly assessed by the tolerance rate of bowel preparation, the incidence of adverse reactions, and the renal function and electrolyte levels before and after bowel preparation.

### 2.4 Statistical methods

A non-inferiority design with two independent sample rates was used in the current study. This study was based on the hypothesis that the success rate of bowel preparation in the 2-L PEG-ELS/Asc group was not inferior to the 3-L PEG-ELS group. Assuming an efficiency rate (p1) of 85% in the study group (the 2-L PEG-ELS/Asc group) and 90% in the control group (the 3-L PEG-ELS group), an absolute difference of less than 9% (non-inferiority threshold Δ) was accepted, according to the formula: n=(Z1-α+Z1 -β)^2^[p1(1-p1)+p2(1-p2)]/(p1-p2+Δ)^2^, we calculated that the sample size of 192 subjects (96 per group) would pass a one-sided proportionality test, providing at the 5% (α) level of significance 90% (1 - β) test efficacy. The SPSS Statistics 25.0 software program (IBM, Armonk, NY, USA) was used to conduct statistical analysis. A Kolmogorov–Smirnov test was used to test the measurement data of normal distribution, described by ± s, and the independent sample t-test was used to compare the two groups. Data with a non-normal distribution were expressed as the median (P25, P75), and were compared using the Mann–Whitney U test. Enumeration data were compared using the chi-square (χ^2^) test. Ordinal data were compared using the Mann–Whitney U test, and *P* < 0.05 indicated a significant difference.

## 3 Results

### 3.1 Baseline data

A total of 240 patients were enrolled in this study, with 120 patients in each group. The 2-L PEG-ELS/Asc group comprised 59 male subjects and 61 female subjects, with an average age of 52 (48, 57) years. Among them, patients with constipation accounted for 37.5% (45/120), and patients with diabetes accounted for 11.7% (14/120). The 3-L PEG group included 60 male subjects and 60 female subjects, with an average age of 54 (47, 60) years. Among them, patients with constipation accounted for 39.2% (47/120), and patients with diabetes accounted for 15.0% (18/120). There was no significant difference in general data such as age, gender, constipation, and diabetes between the two groups (*P* > 0.05) (see **Table 1**).

**Table 1:** Comparison of patients between the two groups [(-x ± s), M(25%,75%)].

Items	2-L PEG-ELS/Asc group n=120	3-L PEG-ELS group n=120	t/Z/*χ* ^2^	*P*
Age	51.88 ± 7.23	53.38 ± 8.01	-1.429	0.153
Gender (male/female subjects)	59/61	60/60	0.017	0.897
Constipation history [n (%)]	45 (37.5%)	47 (39.2%)	0.071	0.791
Diabetes [n(%)]	14 (11.7%)	18 (15.0%)	0.577	0.448

PEG-ELS, polyethylene glycol electrolyte lavage solution; PEG-ELS/Asc, polyethylene glycol electrolyte lavage solution combined with ascorbic acid tablets.

### 3.2 Comparison of the bowel cleansing effects between the two groups

In the 2-L-PEG-plus-Vc group, the total BBPS score was 6.99 ± 1.50 points, with 2.27 ± 0.67 points for the left colon, 2.66 ± 0.49 points for the transverse colon, and 2.07 ± 0.58 points for the right colon. In the 3-L PEG group, the total BBPS score was 7.48 ± 1.30 points, with 2.43 ± 0.60 points for the left colon, 2.83 ± 0.40 points for the transverse colon, and 2.23 ± 0.57 points for the right colon. The BBPS scores of the middle and right colon, and the total BBPS scores between the two groups, showed statistically significant differences (*P* < 0.05). The success rate of bowel preparation in the 2-L PEG-ELS/Asc group was 85.0% (102/120); for 3-L PEG-ELS group, this was 90.8% (109/120), indicating no significant difference between the two groups (*P* > 0.05). For bowel-cleansing grading according to the total BBPS score, there were 46 cases of excellent, 56 cases of good, 6 cases of poor, and 12 cases of very poor in the 2-L PEG-ELS/Asc group. In the 3-L PEG-ELS group, there were 62 excellent cases, 47 good cases, 7 poor cases, and 4 very poor cases. Among these, there were significant differences in the number of patients with bowel cleanliness grades between the two groups (*P* < 0.05) (see **Tables 2**, **3**).

**Table 2:** Comparison of bowel cleansing effects between the two groups [M(25%, 75%)].

BBPS score (points)	2-L PEG-ELS/Asc group n=120	3-L PEG-ELS group n=120	Z/*χ* ^2^	*P*
Left colon	2.27 ± 0.67	2.43 ± 0.60	-1.948	0.052
Transverse colon	2.66 ± 0.49	2.83 ± 0.40	-2.944	0.004
Right colon	2.07 ± 0.58	2.23 ± 0.57	-2.115	0.032
Total score	6.99 ± 1.50	7.48 ± 1.30	-2.704	0.007
Bowel preparation success rate [n (%)]	102 (85.0%)	109 (90.8%)	1.922	0.166

PEG-ELS, polyethylene glycol electrolyte lavage solution; PEG-ELS/Asc, polyethylene glycol electrolyte lavage solution combined with ascorbic acid tablets.

**Table 3:** Comparison of BBPS score grading between the two groups.

BBPS score grading	2-L PEG-ELS/Asc group n=120	3-L PEG-ELS group n=120	*χ* ^2^	*P*
Excellent (n)	46	62	4.310	0.038
Good (n)	56	47	1.378	0.241
Poor (n)	6	7	0.081	0.776
Very poor (n)	12	4	4.286	0.038

PEG-ELS, polyethylene glycol electrolyte lavage solution; PEG-ELS/Asc, polyethylene glycol electrolyte lavage solution combined with ascorbic acid tablets.

### 3.3 Evaluation of the bowel cleansing effect in patients with diabetes and constipation between the two groups

Analysis revealed that the proportion of patients with a history of diabetes and/or constipation in the two patient groups with poor and very poor bowel cleanliness was high (13 cases in the 2-L PEG-ELS/Asc group and 8 cases in the 3-L PEG group). Therefore, based on the presence of a history of diabetes and constipation, the two patient groups were further divided for statistical analysis. The results showed that the success rate of bowel preparation for patients with diabetes and/or constipation in both groups was significantly lower than for patients without diabetes and constipation in the same group (42/55 vs. 60/65 in the 2-L PEG-ELS/Asc group and 45/54 vs. 64/66 in the 3-L PEG group), and the difference was statistically significant (*P* < 0.05). However, there was no significant difference in the success rate of bowel preparation between the two groups (*P* > 0.05) (see **Table 4**).

**Table 4:** Evaluation of bowel cleansing effect of diabetes and constipation patients between the two groups.

	Patients with a history of diabetes and/or constipation	Patients without a history of diabetes and/or constipation	*χ* ^2^	*P*
Bowel preparation success rate in the 2-L PEG-ELS/Asc group (%)	76.7% (42/55)	92.3% (60/65)	5.940	0.015
Bowel preparation success rate in the 3-L PEG-ELS group (%)	83.3% (45/54)	96.7% (64/66)	5.096	0.024

PEG-ELS, polyethylene glycol electrolyte lavage solution; PEG-ELS/Asc, polyethylene glycol electrolyte lavage solution combined with ascorbic acid tablets.

### 3.4 The detection rate of polyps and adenomas in the two groups

The detection rates of total polyps, small polyps (size ≤5 mm), adenomas, left, middle, and right colon polyps in the 2-L PEG-ELS/Asc group were 63.3% (76/120), 52.5% (62/120), 39.2% (47/120), 48.3% (58/120), 21.7% (26/120), and 25.8% (31/120), respectively. The detection rates for total polyps, small polyps (size ≤5mm), adenomas, left, middle, and right colon polyps in 3-L PEG-ELS group were 65.0% (78/120), 55.8% (67/120), 45.8% (55/120), 46.7% (56/120), 23.3% (28/120), and 25.0% (30/120), respectively. There was no significant difference between the two groups (*P* > 0.05) (see **Table 5**).

**Table 5:** Comparison of the detection rates of large intestinal polyps between the two groups.

Items	2-L PEG-ELS/Asc group n=120	3-L PEG-ELS group n=120	*χ* ^2^	*P*
Polyps [n (%)]	76 (63.3%)	78 (65.0%)	0.072	0.788
≤5mm Polyps [n (%)]	63 (52.5%)	67 (55.8%)	0.269	0.604
Adenoma [n (%)]	47 (39.2%)	55 (45.8%)	1.091	0.296
Left colonic polyp [n (%)]	58 (48.3%)	56 (46.7%)	0.067	0.796
Transverse colon polyp [n (%)]	26 (21.7%)	28 (23.3%)	0.096	0.757
Right colonic polyp [n (%)]	31 (25.8%)	30 (25.0%)	0.022	0.882

PEG-ELS, polyethylene glycol electrolyte lavage solution; PEG-ELS/Asc, polyethylene glycol electrolyte lavage solution combined with ascorbic acid tablets.

### 3.5 Adverse reactions and the tolerance assessment of the two groups

The most common adverse reactions in the two groups were abdominal distension, followed by nausea. There was no statistical difference in the incidence of different adverse reactions after a pairwise comparison between the two patient groups (*P* > 0.05). In the 2-L PEG-ELS/Asc group, there were 82 patients with degree-I oral drug tolerance, 30 patients with degree II, and 8 patients with degree III, and the tolerance rate was 93.3% (112/120). In the 3-L PEG-ELS group, there were 61 patients with degree-I oral drug tolerance, 36 patients with degree II, and 23 patients with degree III, and the tolerance rate was 80.2% (97/120). There were statistically significant differences in the number of patients with tolerance degrees I and III, as well as the total tolerance rate after comparison between the two patient groups (*P* < 0.05) (see **Table 6**).

**Table 6:** Comparison of adverse reactions and tolerance between the two groups.

Adverse reactions and tolerance	2-L PEG-ELS/Asc group n=120	3-L PEG-ELS group n=120	*χ* ^2^	*P*
Nausea [n (%)]	12 (10.0%)	18 (15.0%)	1.371	0.242
Vomiting [n (%)]	3 (2.5%)	7 (5.8%)	1.670	0.196
Abdominal distension [n (%)]	22 (18.3%)	31 (25.8%)	1.961	0.161
Abdominal pain [n (%)]	3 (2.5%)	6 (3.0%)	0.462	0.497
Dizziness [n (%)]	2 (1.7%)	4( 3.3%)	0.171	0.679
Tolerance I (n)	82	61	7.630	0.006
Tolerance II (n)	30	36	0.752	0.386
Tolerance III (n)	8	23	8.335	0.004
Tolerance rate (%)	93.3%(112/120)	80.2%(97/120)	8.335	0.004

PEG-ELS, polyethylene glycol electrolyte lavage solution; PEG-ELS/Asc, polyethylene glycol electrolyte lavage solution combined with ascorbic acid tablets.

### 3.6 Changes in renal function and electrolytes before and after bowel preparation in the two groups

The levels of creatinine, serum potassium, serum sodium, serum chlorine, serum calcium, and serum phosphorus in the two groups before and after bowel preparation were all within the normal range. These indicators showed no significant difference before and after the administration of medication (*P* > 0.05) (see **Table 7**).

**Table 7:** Changes in renal function and electrolytes before and after bowel preparation between the two groups [(-x ± s), M (25%-75%)].

Items	Before Bowel Preparation	After Bowel Preparation	t/Z	*P*
Creatinine in the study group (µmol/L)	62 (52,74)	61.5 (51,74.75)	-0.403	0.687
Serum potassium in the study group (mmol/L)	4.0 (3.8,4.3)	4.0 (3.9,4.3)	-0.767	0.443
Serum sodium in the study group (mmol/L)	140 (138.25,141)	140 (138,141)	-0.148	0.883
Serum chlorine in the study group (mmol/L)	104 (102,105.75)	104 (102,106)	-0.326	0.744
Serum calcium in the study group (mmol/L)	2.31 ± 0.10	2.30 ± 0.09	1.761	0.086
Serum phosphorus in the study group (mmol/L)	1.15 ± 0.14	1.14 ± 0.12	1.730	0.086
Creatinine in the control group (µmol/L)	60 (51,72)	60 (52,70.75)	-0.668	0.504
Serum potassium in the control group (mmol/L)	3.95 (3.8,4.1)	4.0 (3.8,4.2)	-0.720	0.471
Serum sodium in the control group (mmol/L)	140 (138,141.75)	140 (139,141)	-0.442	0.658
Serum chlorine in the control group (mmol/L)	104 (103,106)	104 (102,105.75)	-1.749	0.080
Serum calcium in the control group (mmol/L)	2.28 (2.21,2.36)	2.29 (2.20,2.36)	-0.609	0.542
Serum phosphorus in the control group (mmol/L)	1.14 ± 0.15	1.17 ± 0.21	-1.589	0.115

## 4 Discussion

The present study demonstrated that the effectiveness and safety of using 2-L PEG-ELS/Asc in bowel preparation for a colonoscopy in hospitalized patients were not inferior to using 3-L PEG-ELS, but the patient’s tolerance were better. Additionally, the bowel preparation effect of the two schemes for patients with diabetes and constipation is worse than that of those without these conditions. These results indicated that 2-L PEG-ELS/Asc protocol may be a favorable choice for colonoscopy bowel preparation in hospitalized patients but a more appropriate protocol is needed for hospitalized patients with diabetes and constipation.

Colonoscopy is considered an important method for diagnosing colorectal diseases, particularly colorectal cancer. Good bowel preparation quality is essential for the detection of bowel diseases, while low-quality bowel preparation will reduce the accuracy of colonoscopy and increase the missed diagnosis rate of diseases, such as adenomas and colonic polyps (2). As one of many purifying drugs, PEG-ELS is widely used in bowel preparation before conducting a colonoscopy due to its good safety and effectiveness. However, PEG-ELS has poor taste and demands a large intake of liquid, which will cause patient discomfort. Most patients will experience nausea, vomiting, palpitations, and abdominal pain. Once this happens, the compliance and tolerance of patients may be reduced. To improve the acceptability of undergoing a colonoscopy, many clinical workers have attempted to add selected combined adjuvant drugs with PEG-ELS, such as senna leaf, bisacodyl, lactulose, ascorbic acid, and magnesium salt, in the hope of reducing the amount of bowel cleansing solution required and to improve patients’ dependence and tolerance.

Ascorbic acid has good safety and taste and is inexpensive. Once the absorption of high-concentration ascorbic acid sodium in the intestine reaches saturation (the daily intake is more than 1 g), the rest remains in the intestinal cavity, which can act synergistically with PEG as an osmotic laxative. A 2-L PEG-ELS, supplemented with ascorbic acid sodium (2-L PEG-ELS/Asc) is the only low-volume PEG-ELS formulation approved by the United States Food and Drug Administration. Some studies (12–15) have shown that, compared with using PEG-ELS only, when the associated PEG-ELS/Asc composite preparation is used as a laxative, based on ensuring the bowel cleansing effect, it can decrease total liquid intake, increase patient acceptance, and significantly reduce adverse reactions, such as vomiting and abdominal distension, thereby showing better effectiveness and safety. Other studies (16, 17) have shown that the 2-L PEG-ELS/Asc alternative outperformed the standard PEG option in terms of the bowel cleansing effect, particularly for the right colon. Due to the lack of corresponding composite preparations in China, the 2-L PEG-ELS/Asc protocol was used in this study to compare the bowel cleansing effect with the 3-L option. The results showed that the success rate of bowel preparation in the 2-L PEG-ELS/Asc group was not inferior to that in the 3-L PEG-ELS group. Moreover, compared with patients in the 3-L PEG-ELS group, although the incidence of adverse reactions in the bowel preparation process of patients in the 2-L PEG-ELS/Asc group was not significantly reduced, their tolerance was significantly improved. Additionally, the detection rates of polyps, small polyps (≤5 mm), and adenomas in the two groups were essentially similar, with no significant statistical difference.

The study showed (18, 19) that diabetes and constipation were risk factors that affected the quality of bowel preparation. For patients who were diabetic/constipated, the bowel preparation effect of using PEG only was often not ideal. The current study found that the proportion of patients with diabetes and constipation was high among patients whose bowel preparation was unqualified. The results of additional statistical analysis showed that the effects of the 2-L PEG-ELS/Asc option and the 3-L PEG option in the bowel preparation of patients with diabetes and/or constipation were much worse than for patients without diabetes, and who were not constipated.

In recent years, it has been reported (20, 21) that patients receiving PEG-ELS/Asc for bowel preparation have a risk of acute kidney injury and electrolyte disturbance. However, in this study, the levels of serum creatinine, serum potassium, serum sodium, serum chlorine, serum calcium, and serum phosphorus in the two groups before and after bowel preparation were within the normal range and showed no statistical difference between the two groups. Additionally, no significant difference was found in the incidence of different adverse reactions after a pairwise comparison between the two patient groups. These results indicated the safety of using PEG-ELS/Asc for bowel preparation. Notably, the most common adverse reactions in the two groups were abdominal distension and nausea. Similar to this finding, Woo et al. also revealed that the main adverse events of PEG/Asc for intestinal preparation were nausea and abdominal distention (22). Several reports have demonstrated that gum chewing had a positive effect on abdominal pain and nausea caused by PEG-ELS, and it may be recommended for patients (23–25).

It is undeniable that this study has some limitations. First, patients were not asked in advance to discontinue medicines such as prokinetics, anti-spasmodic agents, or sedatives that may affect intestinal motility. Second, this study was a single-center study, no second blinded video or photo analysis of colon by external physician. Third, this study could not provide more detailed information regarding other variables that could affect the quality of a bowel preparation, such as narcotic use, indications for the procedure, BMI and constipation grading. Additional studies are required for more precise assessment.

In summary, the 2-L PEG-ELS/Asc option was not inferior to the 3-L PEG-ELS option in terms of its bowel cleansing effect; it was also better tolerated by patients. The incidences of adverse reactions were similar between the two options, and there were no differences in the detection rates of polyps, small polyps (size ≤5mm), and adenomas, diminishing the risk of renal function damage and electrolyte disturbance. However, the effects of the 2-L PEG-ELS/Asc option and 3-L PEG-ELS options in the bowel preparation of patients with diabetes and constipation were not ideal. Therefore, for general patients, the 2-L PEG-ELS/Asc option can be promoted as an excellent bowel preparation method, while for patients with diabetes and constipation, more appropriate and effective bowel preparation options must be explored.

## Data availability statement

The original contributions presented in the study are included in the article/supplementary material. Further inquiries can be directed to the corresponding author.

## Ethics statement

The studies involving human participants were reviewed and approved by Ethics Committee of Wenzhou People’s Hospital (No. 2020-181). The patients/participants provided their written informed consent to participate in this study.

## Author contributions

Conception and design of the research: LCW, EDZ, and XXL. Acquisition of data: HYS and XZL. Analysis and interpretation of the data: HYS and XZL. Statistical analysis: JYP. Obtaining financing: LCW. Writing of the manuscript: LCW, EDZ, and XXL. Critical revision of the manuscript for intellectual content: LCW and XXL. All authors contributed to the article and approved the submitted version.
